# Online Traffic Crash Risk Inference Method Using Detection Transformer and Support Vector Machine Optimized by Biomimetic Algorithm

**DOI:** 10.3390/biomimetics9110711

**Published:** 2024-11-19

**Authors:** Bihui Zhang, Zhuqi Li, Bingjie Li, Jingbo Zhan, Songtao Deng, Yi Fang

**Affiliations:** 1 School of Naval Architecture and Ocean Engineering, Jiangsu University of Science and Technology, Zhenjiang 212003, China; 2 School of Computer and Control Engineering, Northeast Forestry University, Harbin 150040, China; 3 College of Fisheries, Guangdong Ocean University, Zhanjiang 524088, China; 4 Information and Communication Engineering, Hainan University, Haikou 570228, China; 5 School of Electronic Information and Electrical Engineering, Shanghai Jiao Tong University, Shanghai 200240, China

**Keywords:** traffic crash risk, mobile robot, TAR-DETR, WOA-SA-SVM, biomimetic algorithm, machine learning

## Abstract

Despite the implementation of numerous interventions to enhance urban traffic safety, the estimation of the risk of traffic crashes resulting in life-threatening and economic costs remains a significant challenge. In light of the above, an online inference method for traffic crash risk based on the self-developed TAR-DETR and WOA-SA-SVM methods is proposed. The method’s robust data inference capabilities can be applied to autonomous mobile robots and vehicle systems, enabling real-time road condition prediction, continuous risk monitoring, and timely roadside assistance. First, a self-developed dataset for urban traffic object detection, named TAR-1, is created by extracting traffic information from major roads around Hainan University in China and incorporating Russian car crash news. Secondly, we develop an innovative Context-Guided Reconstruction Feature Network-based Urban Traffic Objects Detection Model (TAR-DETR). The model demonstrates a detection accuracy of 76.8% for urban traffic objects, which exceeds the performance of other state-of-the-art object detection models. The TAR-DETR model is employed in TAR-1 to extract urban traffic risk features, and the resulting feature dataset was designated as TAR-2. TAR-2 comprises six risk features and three categories. A new inference algorithm based on WOA-SA-SVM is proposed to optimize the parameters (C, g) of the SVM, thereby enhancing the accuracy and robustness of urban traffic crash risk inference. The algorithm is developed by combining the Whale Optimization Algorithm (WOA) and Simulated Annealing (SA), resulting in a Hybrid Bionic Intelligent Optimization Algorithm. The TAR-2 dataset is inputted into a Support Vector Machine (SVM) optimized using a hybrid algorithm and used to infer the risk of urban traffic crashes. The proposed WOA-SA-SVM method achieves an average accuracy of 80% in urban traffic crash risk inference.

## 1. Introduction

Traffic crashes have become a critical global public safety issue, causing significant casualties annually. The World Health Organization’s (WHO) 2018 Global Report on Road Safety states that around 1.35 million individuals globally succumb annually to traffic crashes [1]. Among those aged 5 to 29, vehicular crashes rank as the primary reason for mortality. Therefore, it is imperative to implement effective preventive measures to reduce traffic crashes. With technological advancements, various AI-driven technologies, including deep learning and machine learning algorithms, are being developed and applied to predict urban traffic collision risks.

As Artificial Intelligence and computer vision technologies continue to progress rapidly, various effective algorithms have been designed and applied for detecting objects. The YOLO (You Only Look Once) algorithm, renowned for its high processing speed [2,3], is extensively applied in real-time traffic monitoring systems. By processing the entire image in one pass, the algorithm efficiently detects cars and people, making it highly suitable for monitoring both highway and urban traffic. Liang et al. [4] employed YOLO v7 [5] to detect vehicles and pedestrians in traffic scenes. Wang et al. [6] employed YOLO v8 [7] to detect infrared targets in nighttime traffic scenes. Zhao et al. [8] employed YOLO v8 to detect Unmanned Aerial Vehicles (UAVs) in aerial images of complex ground scenes. Khalili and Smyth [9] employed YOLOv8 for target detection in aerial imagery and traffic scenes. However, these algorithms enhance detection accuracy by increasing the complexity and size of the network models. On the other hand, using a lightweight model results in lower accuracy detection. Furthermore, their efficiency in harsh light environments, like nighttime or dimly lit situations, requires enhancement. New algorithms, including DETR (Detection Transformer) and RT-DETR (Real-Time Detection Transformer), utilize the Transformer framework and have shown impressive efficacy in identifying targets. DETR removes superfluous processes in conventional techniques by directly detecting the positions and types of objects in images, yet its training method is intricate and demands substantial computational power. RT-DETR fine-tunes DETR to enhance its speed and effectiveness in real-time application situations. Xia et al. [10] employed an improved DETR for traffic sign detection in autopilot systems. Yu and Shin [11] employed RT-DETR for target detection in UAV images. Liu et al. [12] employed RT-DETR to detect safety helmets in foggy weather. Additionally, there are several challenges associated with traffic crash risk data extraction using object detection technology, such as (1) monitoring a moving vehicle in a traffic situation with mixed traffic; (2) dealing with road user shadows and tracking vehicle platoons [13]; (3) because it necessitates constantly keeping watch of every person, evaluating the likelihood of a collision [14]. The trajectory-based approach provides higher accuracy using vehicle tracking, drone data, and image processing tools. Also, the automated detection tools of moving objects can improve pedestrian safety at intersections [15].

Real-time traffic crash risk inference models have attracted significant attention in road safety analysis. The fundamental principle of these models is to associate the likelihood of accidents occurring on roadways or in proximity to vehicles with the monitored traffic conditions. Wang et al. [16] utilize geometric data, Microwave Vehicle Detection System (MVDS) data, and weather data to investigate the likelihood of crashes. Ren and Xu [17] consider various factors, such as time, weather, visibility, and vehicle data, to better understand highway-rail grade crossing crashes. Li et al. [18] analyze traffic flow characteristics, signal timing, and weather conditions to predict crash risk. The methods mentioned above primarily rely on static data and traditional perception techniques, lacking the capacity to fully capture real-time traffic dynamics and complex traffic environments. These methods fail to effectively respond to complex road scenarios, unexpected traffic behaviours, or dynamic changes and do not fully leverage modern vision or sensor data for real-time responses and accurate analysis. Among the existing studies about real-time crash risk inference, most are limited to freeways [19,20,21] rather than urban arterials [22]. The traffic environment of arterials is much more complicated than freeways due to the existence of the intersections. Thus, it is difficult to predict arterial crash risk only based on traffic flow parameters; signal timing needs to be considered as well [23]. Furthermore, traffic crash risk inference is a typical classification problem since its output is a categorical event.

Typically, real-time crash risk inference relies on two primary approaches: statistical methods and machine learning techniques. In particular, statistical approaches encompass models such as conditional logit, log-linear, and logistic regression, among others. These models typically rely on matched case–control data and are based on strong assumptions. Considering these limitations, machine learning methods become popular, such as Support Vector Machine (SVM), etc. Ahmed et al. [24] used various machine learning models, such as Random Forest (RF), Decision Jungle (DJ), AdaBoost, XGBoost, Light Gradient Boosting Machine (L-GBM), and Categorical Boosting (CatBoost) to analyze New Zealand traffic accident data from 2016 to 2020. Gan et al. [25] employed the Deep Forest algorithm to predict the severity of traffic accidents. Dong et al. [26] employed the Multivariate Negative Binomial (MVNB) model to predict traffic accidents. Yang et al. [27] employed the Random Forest algorithm to predict the severity of traffic accidents using data from the China National Automobile Accident dataset. Gataric et al. [28] employed artificial neural networks to predict traffic accidents on public roads in the Republic of Serbia and Republika Srpska (Bosnia and Herzegovina). Aldhari et al. [29] employed Random Forest, XGBoost, and logistic regression to predict the severity of highway traffic accidents in Saudi Arabia. Samerei and Aghabayk [30] employed a random parameters logit model, interpretable machine learning, and clustering to analyze the transition from two-vehicle collisions to chain-reaction crashes. These studies generally emphasize the importance of identifying the various factors contributing to traffic accidents and highlight the role of machine learning in advanced accident analysis. Ultimately, despite the significant advancements in traffic safety analysis brought about by current research methods, there is still a need for ongoing enhancements.

To address the insufficient accuracy of current object detection technology and enhance machine learning’s capability to predict traffic crash risk, this article introduces an automatic inference method of traffic crash risk based on TAR-DETR and optimizing SVM model with biomimetic algorithm. The object detection model is based on an improved RT-DETR. Not only does it maintain high accuracy, but it is particularly effective at identifying cars and people. The SVM model optimized by the biomimetic algorithm can more accurately infer traffic crash risks and more effectively identify potential risks.

The primary results of this research are as follows:

1. To better train and test the effectiveness of deep learning models in real and complex traffic environments, and to address data scarcity challenges, we used infrared imaging devices in real road environments to construct a high-quality urban traffic object detection dataset named TAR-1. Additionally, we incorporated traffic crash images from Russian news.
2. In this study, we self-developed the TAR-DETR object detection model. To enhance detection accuracy, we introduced the Context-Guided Reconstruction Feature Network (CGRFN). We designed RCM for spatial feature reconstruction and context extraction, capturing global context to model key regions. We utilized the PCE module to integrate different feature levels, enhancing the model’s context awareness. Additionally, we designed FBM and DIF modules for multi-scale feature fusion. The modules enhance the model’s extraction of features and its capacity to identify objects amidst intricate backdrops.
3. In this paper, we employ the bionic swarm intelligence optimization algorithm (Whale Optimization Algorithm and Simulated Annealing Algorithm) to optimize the parameters (C, g) of the SVM, thereby enhancing its ability to infer traffic crash risk.

The outline of this paper is as follows: Section 2 presents a detailed analysis of the contents of the two datasets (TAR-1 and TAR-2) used in the present study. Section 3 describes the proposed method. Section 4 presents experimental results and analysis of the extracted features. Section 5 discusses the potential implications and drawbacks. Section 6 summarizes the conclusions.

## 2. Datasets

### 2.1. TAR-1: Urban Traffic Objects Dataset

In this study, we released for the first time a dataset of urban traffic objects in two parts: Part A was acquired using an infrared sensing camera in real road environments, and Part B was extracted from Russian news videos. Initially, our primary consideration in searching for a traffic crash dataset was to use a dataset from China exclusively. However, Chinese traffic crash data may contain mosaics in key feature areas, which could significantly hinder TAR-DETR’s feature recognition. As a result, we decided to incorporate Russian traffic crash data.

**Figure 1:**
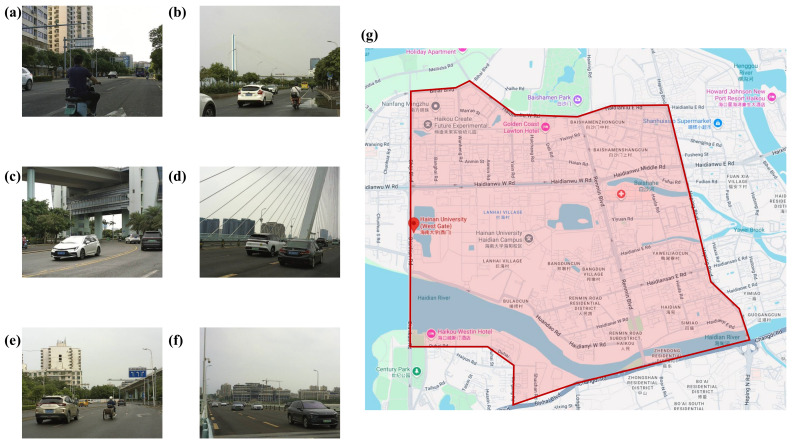
Example of TAR-1 dataset and recording data area. (**a**–**f**) Example images; (**g**) recording data area.

Part A was collected from 9 a.m. on 23 June 2024, to 8 p.m. on 28 June 2024, in the main road area around People’s Avenue in the Meilan District of Haikou City, Hainan Province, China. As depicted in Figure 1g, the data area extends from Bihai Blvd in the north to Changdi Road in the south, and from Diankun Road in the west to Heping Road in the east. The images categorize targets into two groups: “people” and “car”. We selected images featuring high car traffic density, people in the road centre, risky positioning of people and cars, complex intersections, and lightly trafficked road sections.

**Figure 2:**
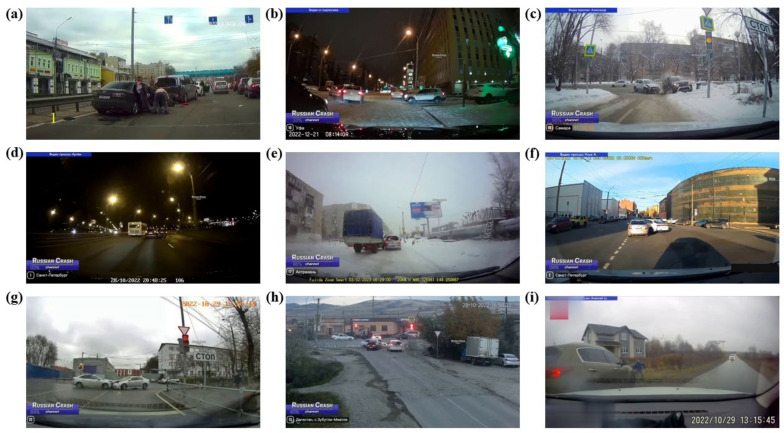
Example of TAR-2. (**a**–**i**) Example collision images.

Part B was derived from Russian news videos featuring car crashes, with still images extracted every 10 frames. The images were categorized into two groups: “people” and “car”. As depicted in Figure 2a–i, we selected images featuring high car traffic density, people in the road centre, risky positioning of people and cars, complex intersections, and lightly trafficked road sections. Additionally, scenes depicting collisions from traffic crashes have been specifically included. These scenarios were selected to provide a comprehensive and challenging collection of realistic traffic conditions, including severe traffic congestion, dangerous interactions between people and cars, and varied road layouts. This not only increases the complexity of the dataset but also boosts its practical value for tasks like target detection and risk inference.

The MixUp is a straightforward linear transformation of the input data to generate a new mixture of samples and corresponding person labels. Briefly, for any two input data label pairs, (*x*_i_, *y*_i_) and (*x*_j_, *y*_j_), MixUp can be described as [31]:

(1) $$\lambda =Beta\left(\alpha ,\alpha \right)$$

(2) $$\tilde{x}=\lambda \cdot x_{i}+\left(1-\lambda \right)\cdot x_{j}$$

(3) $$\tilde{y}=\lambda \cdot y_{i}+\left(1-\lambda \right)\cdot y_{j}$$

where $\lambda =Beta\left(\alpha ,\alpha \right)$ represents a scale parameter *λ* drawn from a *Beta* distribution, the *Beta* distribution has a shape parameter *α*. The *Beta* distribution is a continuous probability distribution defined on the interval [0, 1]. $\tilde{x}$ and $\tilde{y}$ are the generated mixture samples and corresponding labels.

**Figure 3:**
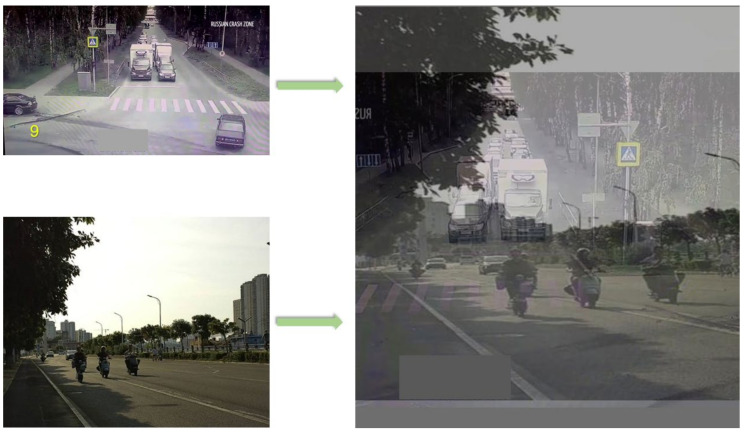
MixUp technology implementation process.

As depicted in Figure 3, the model’s generalization ability is enhanced through the generation of new training samples using MixUp. This technique involves creating weighted linear combinations of two images along with their corresponding labels, a common method for data augmentation. Specifically, we blend images from Part A and Part B in a specific ratio, effectively merging the visual features of different scenes at the pixel level and integrating the target categories at the label level. Additionally, it boosts the model’s robustness in handling challenging situations like occlusions, lighting variations, and congestion. Utilizing MixUp, models demonstrate enhanced accuracy and stability in target detection and risk prediction. The TAR-1 dataset, originally comprising 1665 labelled images, was expanded to 3000 images through the application of MixUp technology.

### 2.2. TAR-2: Urban Traffic Crash Risk Features Dataset

Sarkar et al. [32] provide an overview of alternative Surrogate Safety Measures (SSM), which have the potential to shed light on the sequence of events leading up to a collision, its root causes, and consequences. The review considers SSM analysis based on traffic video data as the most common approach. However, monitoring moving vehicles with high accuracy in complex traffic environments and the need for more precise detection models have become challenges limiting SSM data extraction. Muktar and Fone [33] analyzed traffic accidents in Montreal by examining factors such as the number of light cars and trucks, people, and emergency cars involved. The variable “Severity” was categorized into five distinct groups: “Damage Below Reporting Threshold”, “Only Property Damage”, “Minor”, “Serious”, and “Fatal”. Le et al. [34] investigated the characteristics of urban traffic accidents in Hanoi, Vietnam, focusing on variables such as vehicle type, accident type, road type, speed limit, and number of victims. Potential problems may arise if observed crashes are used, especially when the data are conditioned on the occurrence of accidents. This potential selectivity can complicate the interpretation of parameters, especially for weather-related factors and certain motorway driving styles (e.g., motorcyclists are more likely to self-select in rain and snow, as discussed in Mannering [35]). More importantly, models based on traditional police data or other real-time data may produce inaccurate predictions if they fail to account for factors that alter the self-selectivity of road users in bad weather or unsafe routes. Therefore, we propose a simpler method to directly extract features from real-time images for crash risk inference.

**Figure 4:**
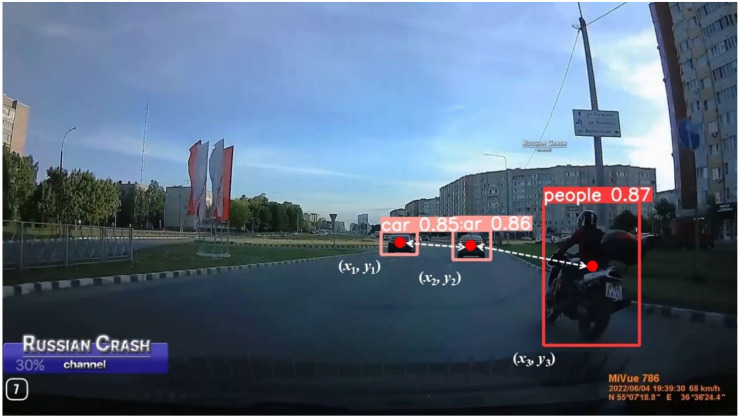
Methods for extracting distances between targets.

Sun et al. [36] employed YOLO to process real-time video images from a camera, initially identifying cars and subsequently determining the pixel size w′ of the vehicle’s anchor frame in the imaging plane. These data were used to calculate the distances between vehicles on the left and right. Building on these methods, this paper introduces a straightforward and effective risk feature extraction technique illustrated in Figure 4. Using TAR-DETR, the coordinate information of the target’s a priori frame is extracted, and its centre point is used to compute the distance to the neutral point of another target. This calculation determines the shortest distances between cars, as well as between cars and humans, as depicted in Equations (4) and (5) as follows:

(4) $$d_{car}=\sqrt{{\left(x_{1}-x_{2}\right)}^{2}+{\left(y_{1}-y_{2}\right)}^{2}}$$

(5) $$d_{people}=\sqrt{{\left(x_{1}-x_{3}\right)}^{2}+{\left(y_{1}-y_{3}\right)}^{2}}$$

where (*x*_1_, *y*_1_) are the coordinates of car_1_, (*x*_2_, *y*_2_) are the coordinates of car_2,_and (*x*_3_, *y*_3_) are the coordinates of people.

In this document, we further develop our criteria for selection by incorporating expertise in the field and assessing the quality of the data. Our goal is to identify the most valuable features to maintain objectivity in our model. The last features employed in our model consist of the total number of vehicles (per picture), total number of people (per picture), shortest distance between cars, shortest distance between people and cars, and confidence level. The target variable “Severity” is categorized into three levels: “Safety”, “Dangerous”, and “Collision”. As indicated in Table 1, the dataset comprises 1665 rows and 6 columns. The diverse nature of this data type indicates that it contains substantial information.

## 3. Methods

**Figure 5:**
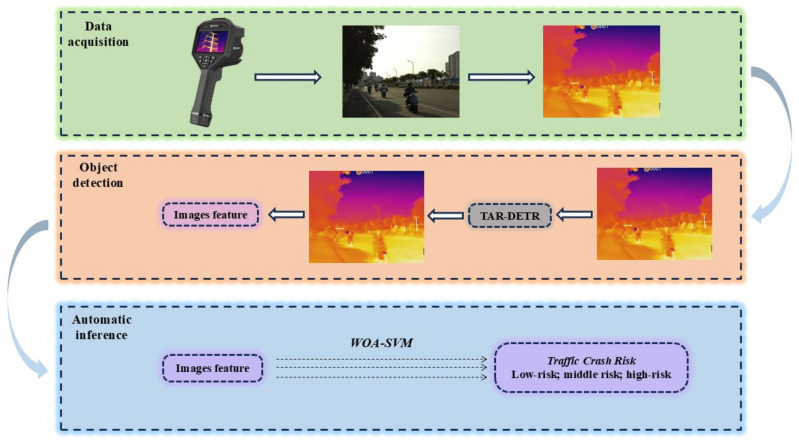
Traffic crash risk inference framework: data acquisition, object detection, and automatic inference.

Figure 5 depicts the structure of the traffic risk inference approach put forward in this study. The approach comprises three components: data collection, object recognition, and automated inference. The data acquisition process has been described in Section 2. In this paper, we extract features from the traffic environment image using the TAR-DETR model and integrate them into a dataset (TAR-2), which is then inputted into the WOA-SA-SVM model to complete the automatic inference process.

### 3.1. Urban Traffic Objects Detection Based on TAR-DETR

**Figure 6:**
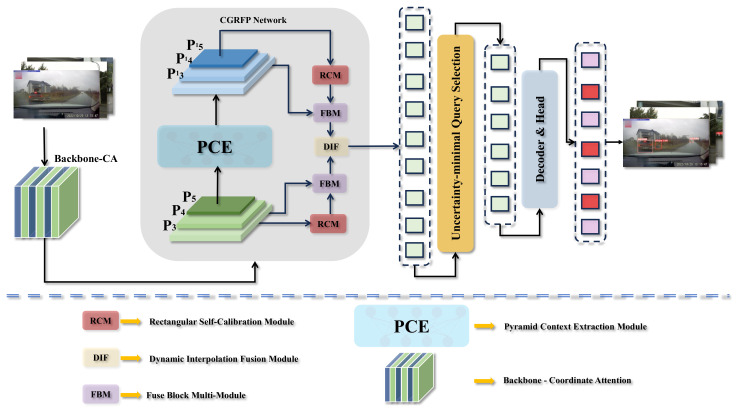
The overall network framework of TAR-DETR encompasses four principal modules: the Rectangular Self-Calibration Module, the Dynamic Interpolation Fusion Module, the Fuse Block Multi-Module, and the Pyramid Context Extraction Module. A mechanism for coordinated attention is incorporated into the backbone network. P_3_, P_4_, and P_5_ represent feature maps derived from disparate levels of the backbone network.

To address the reduced accuracy of people and car detection models in urban traffic with multiple object sources, we designed and integrated a series of optimization methods to develop an object detection network for urban traffic: the Context-Guided Reconstruction Feature Network (CGRFN). As depicted in Figure 6, this section describes the overall framework of TAR-DETR and the optimization methods (CGRFN, Pyramid Context Extraction Module, Fuse Block Multi-Module, and Dynamic Interpolation Fusion Module).

#### 3.1.1. Context-Guided Reconstruction Feature Network

To overcome the limitations of the original RT-DETR network in detecting small targets in complex urban traffic environments, this study develops a Context-Guided Reconstruction Feature Network (CGRFN) in the Neck section, which is specifically designed to process image information across multiple dimensions and optimize object detection performance through dynamic feature fusion and multiscale processing techniques. The design of this network architecture reflects the need for accurate and efficient processing, particularly for challenging small object detection scenarios in urban traffic environments.

**Figure 7:**
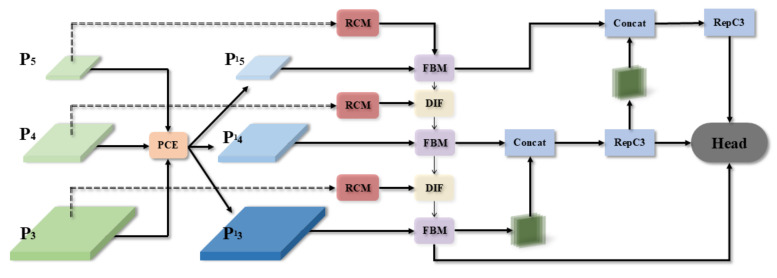
The overall network framework of CGRFN.

As depicted in Figure 7, the Pyramid Context Extraction (PCE) module first extracts rich contextual information from the base feature maps P_3_, P_4_, and P_5_, forming three different scales of feature maps: P′_3_, P′_4_, and P′_5_. Next, indexing operations are used to individually obtain the output of these extracted feature maps, followed by further feature enhancement using the Rectangular Self-Calibration Module (RCM). In particular, for the P_5_ layer, Fuse Block Multi-Module (FBM) is used to fuse the output of the RCM with the original P’5; for the P_4_ and P_3_ layers, along with FBM, Dynamic Interpolation Fusion (DIF) is introduced to optimize the integration of features at different scales. Additional convolutional processing is applied to the features in layers P_3_ and P_4_ using a 3 × 3 kernel to refine feature representation and progressively reduce the spatial dimensionality from 256 to 128, and finally to 64 dimensions. This process effectively filters out less informative features, focusing on those critical for detection. The processed feature maps undergo concatenation and convolutional processing via the RepC3 module to further refine and symmetrize the features. Finally, these feature maps are passed through the RT-DETR head for real-time object detection.

#### 3.1.2. Pyramid Context Extraction Module

**Figure 8:**
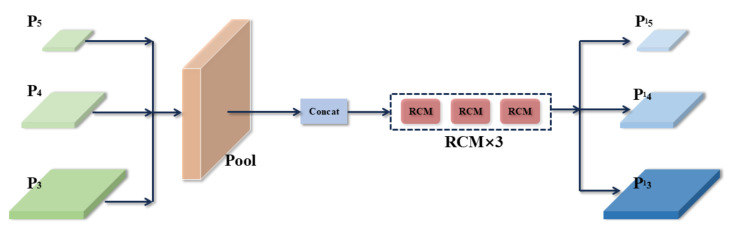
The network framework of the PCE.

To address the insufficient recognition capability in complex urban traffic environments, this paper proposes a new method that integrates the PCE module into the Neck network, as depicted in Figure 8. Specifically, the PCE module enhances the model’s context awareness by integrating feature information from different layers. The main inputs to the PCE are derived from P3, P4, and P5 in the feature pyramid. The fused feature maps are processed through a pool layer and then integrated via concatenation before being passed to the RCM. Subsequently, the feature representation of P3, P4, and P5 is further enhanced using three RCMs. These modules effectively address issues such as scale variation, occlusion, and complex background interference in multi-object recognition scenarios, thereby improving the model’s robustness and recognition accuracy in congested urban traffic environments.

#### 3.1.3. Rectangular Self-Calibration Module

**Figure 9:**
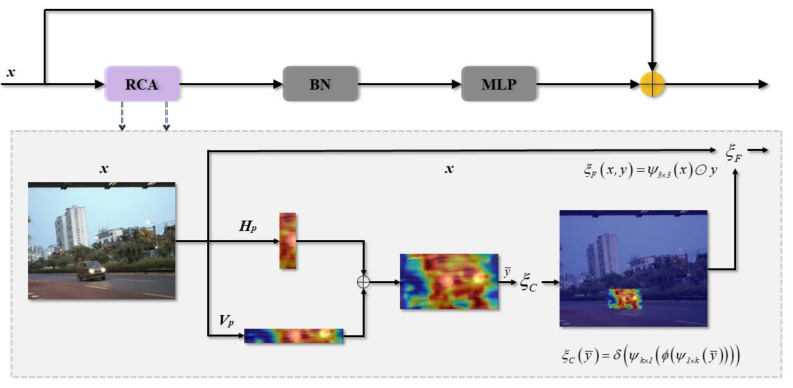
The network framework of RCM.

The Rectangular Self-Calibration Module (RCM) is designed to keep the model focused on the foreground while capturing the axial global context for pyramidal context extraction. As shown in Figure 9, the module comprises rectangular self-calibration attention, batch normalization, and a multilayer perceptron (MLP). Rectangular self-calibrating attention (RCA) uses horizontal and vertical pooling to capture the axial global context in both directions, generating two distinct axis vectors. By summing these vectors using broadcast addition, RCA efficiently models rectangular regions of interest. A shape self-calibration function is then designed to align the region of interest more closely with the foreground object. Two large kernel strip convolutions are applied to calibrate the attention maps in both horizontal and vertical directions. First, horizontal strip convolution is used to adjust each row of elements, aligning the horizontal shape more closely with the foreground object. The features are then normalized using Batch Normalization (BN), and nonlinearity is added with Rectified Linear Unit (ReLU). Next, vertical bar convolution is applied to calibrate the shape in the vertical direction. This decouples the convolutions in both directions to fit any shape. Figure 9 visualizes how the rectangular self-calibrating attention effectively models the key rectangular region, adjusting the features to focus on the important areas.

The shape self-calibration function [37] can be formulated as follows:

(6) $$\xi C\left(\bar{y}\right)=\delta \left(\psi_{k\times 1}\left(\phi \left(\psi_{k\times 1}\left(\bar{y}\right)\right)\right)\right)$$

where *ψ* denotes the convolution operation using a large kernel, and *k* indicates the size of the kernel used in strip convolution. *ϕ* indicates that Batch Normalization is applied, followed by the ReLU activation function, and *δ* indicates the application of the Sigmoid activation function.

In addition, a feature fusion function [36] is designed to fuse attention features with input features.

(7) $$\xi F\left(x,y\right)=\psi_{3\times 3}\left(x\right)⊙y$$

where *ψ*_3×3_ indicates the depthwise convolution operation with kernel 3 × 3. *y* represents the attention feature extracted from the previous step. ⊙ denotes the Hadamard product.

The structure of the RCM is illustrated in Figure 9. The following describes it [38]:

(8) $$F_{out}=\rho \left(\xi F\left(x,\xi C\left(H_{p}\left(x\right)⊕V_{p}\left(x\right)\right)\right)\right)+x$$

where ⊕ represents the broadcast addition operation. *H_P_* and *V_P_* represent the operations of Horizontal Pooling and Vertical Pooling. *ρ* refers to BN and MLP.

#### 3.1.4. Fuse Block Multi and Dynamic Interpolation Fusion Modules

Since dangerous events in urban traffic environments may also occur at the edges of an image, it is crucial to extract information from these regions as well. Furthermore, since the prediction of dangerous events in this paper relies on feature extraction with high confidence, an extremely high confidence level is necessary to enhance the dataset quality. To address the need for high confidence, the Fuse Block Multi and Dynamic Interpolation Fusion modules are proposed to further fuse multi-scale features. These modules enhance the model’s multi-scale feature representation and improve its recognition of objects in complex backgrounds through dynamic interpolation and multi-feature fusion.

**Figure 10:**
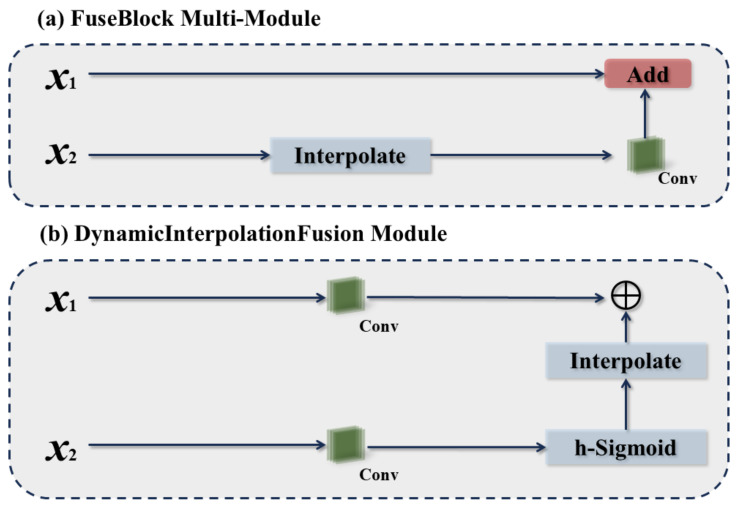
The network framework of FBM and DIF modules. (**a**) FBM; (**b**) DIF module.

As depicted in Figure 10, *x*_1_ and *x*_2_ represent the information passing through the PCE module and the RCM, respectively. In the FBM, after the same information is extracted from the features by different modules, *x*_2_ is interpolated to match the numerical shape of *x*_1_, and then summed with *x*_1_ following a convolution operation. In the DFM module, after extracting the same information from the features using different modules, *x*_1_ and *x*_2_ are each convolved once. After convolution, *x*_2_ undergoes another convolution with an activation function and interpolation before being multiplied by the convolved *x*_1_.

### 3.2. Urban Traffic Crash Risk Inference Method Based on WOA-SA-SVM

Recently, many studies have assessed the effectiveness of various machine learning models in forecasting the severity of traffic crashes. Samerei and Aghabayk [39] employed classification and regression trees (CART), K-nearest neighbours (KNNs), random forest (RF), artificial neural networks (ANN), and Support Vector Machines (SVMs) to analyze crash severity. Their study aimed to implement interpretable machine learning to visualize the impacts of factors on crash severity using five years of freeway data from Iran. Yang et al. [40] employed K-Nearest Neighbor classification (KNN), SVM, and Integrated Decision Tree (EDT) models to predict pedestrian fatalities, and the results indicated that the SVM and EDT models outperformed the KNN model in terms of accuracy. Lu et al. [41] employed the LS-SVM algorithm for forecasting real-time area risk on a lane-by-lane basis, demonstrating that the LS-SVM model strongly supports the development of personalized active traffic control strategies for different lanes. Therefore, this paper used the SVM algorithm, which is fast to train and has strong generalization capability.

SVM offers rapid training and strong generalization capabilities. It exhibits enhanced robustness and generalization performance when dealing with nonlinear data and small datasets. However, the parameters g and C of SVM greatly influence the classification accuracy. There are problems, such as slow convergence speed and premature convergence accuracy, when a single optimization algorithm is used to optimize (C, g). The hybrid swarm intelligence optimization algorithm can use the advantages of different algorithms to complement each other. Oladejo et al. [42] propose the Hiking Optimization Algorithm (HOA), inspired by hiking, a popular recreational activity, due to the similarity between the search landscapes of optimization problems and the mountainous terrains navigated by hikers. Khunkitti et al. [43] propose the Two-Archive Harris Hawk Optimization (TwoArchHHO) to solve Many-oObjective Optimal Power Flow (MaOOPF) problems. Wang et al. [44] propose a novel Artificial Protozoa Optimizer (APO) that is inspired by protozoa in nature. Therefore, a new hybrid swarm intelligence optimization algorithm, WOA-OA, is proposed for traffic crash risk inference.

#### 3.2.1. Support Vector Machine Approach

Support Vector Machine Approach (SVM) [45] is a machine learning model used for both regression and classification tasks. It is based on the principles of machine learning, statistics, and optimization. The model’s performance is evaluated using a measure of fitness, which is determined by empirical error and the hypothesis space. A detailed description of the algorithm’s steps and mathematical formulations is provided in Appendix B.

#### 3.2.2. Whale Optimization Algorithm

The Whale Optimization Algorithm (WOA) [46] is a biomimetic optimization technique that mimics the hunting behaviour of humpback whales. This algorithm mimics the bubble net feeding strategy of whales, in which prey is trapped through coordinated spirals as whales encircle them. WOA has shown strong performance in various optimization problems due to its global search ability and robustness. A detailed description of the algorithm’s steps and mathematical formulations is provided in Appendix C.

#### 3.2.3. Simulated Annealing Algorithm

The Simulated Annealing (SA) [47] algorithm is inspired by the annealing process in metallurgy, where materials are heated and gradually cooled to eliminate defects. In optimization, SA employs a similar approach, where solutions are explored iteratively and accepted or rejected based on a temperature-dependent probability function. The algorithm allows the exploration of the solution space by occasionally accepting worse solutions, thus avoiding local minima and facilitating a more global search. A detailed description of the algorithm’s steps and mathematical formulations is provided in Appendix D.

#### 3.2.4. Hybrid Bionic Intelligent Optimization Algorithm

**Figure 11:**
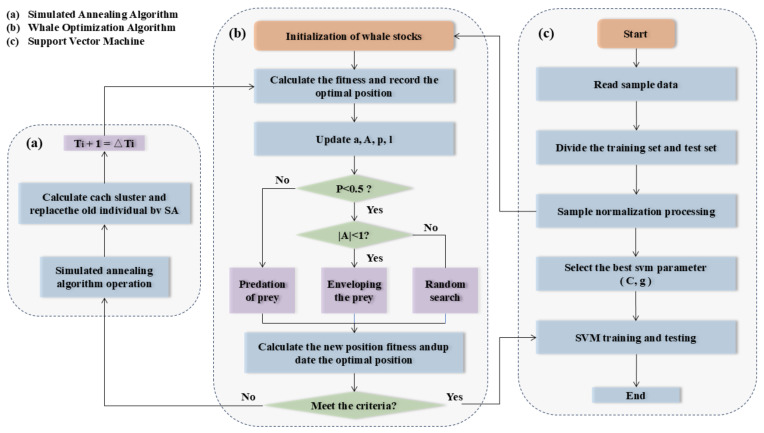
The framework of WOA-SA-SVM. (**a**) SA algorithm; (**b**) WOA; (**c**) SVM algorithm.

As illustrated in Figure 11, the Hybrid Bionic Intelligent Optimization Algorithm integrates the strengths of various optimization techniques, including Simulated Annealing (SA) and Whale Optimization Algorithm (WOA). This hybrid approach combines SA’s ability to avoid local minima with WOA’s global search capability to enhance convergence and solution quality. The optimized parameters are then fed back into the SVM. A detailed, step-by-step description and algorithmic flow of algorithm are provided in Appendix E.

## 4. Results and Analysis

### 4.1. Evaluation of Detection Performance of TAR-DETR

The training and testing of all models were conducted on a single computer with an Intel(R) Core (TM) i7-12700k CPU and an NVIDIA GeForce RTX 3090 24 GB GPU. The experiments utilized PyTorch version 1.12.1, with Python 3.8.16 as the programming language.

This study employed precision, recall, and Average Precision (AP) to assess the performance of each model [2]. Precision is the probability of being a positive sample out of all the samples predicted to be positive. It represents the precision of predicting the outcome of a positive sample. A positive sample refers to an image region in the model’s predicted results, where the Intersection over Union (IoU) with the ground truth bounding box labelled by the researchers exceeds a threshold. Recall is the probability of being predicted as a positive sample among the actual positive samples. Precision and recall have opposite tendencies, and when precision increases, the value of recall often decreases. The PR (Precision-Recall) curve represents the relationship between precision and recall. Average Precision (AP) refers to the area under the PR curve. The formulas for precision, recall, and AP are shown below. AP_i_ represents the value of AP when the IoU is equal to *i*. mAP refers to the mean value of AP. GIoU (Generalized Intersection over Union) has an additional “Generalized” compared to IoU.

(9) $$precision=\frac{TP}{TP+FP}$$

(10) $$recall=\frac{TP}{TP+FN}$$

(11) $$AP=\int_{1}^{0}precision\left(recall\right)d\left(recall\right)$$

(12) $$mAP=\frac{\sum_{1}^{N}AP}{N}$$

(13) $$mAP_{50:90}=\frac{1}{10}\left(mAP_{50}+mAP_{55}+\cdots +mAP_{95}+mAP_{55}\right)$$

(14) $$GIoU=IoU-\frac{\left|C-\left(A\cap B\right)\right|}{C}$$

where True Positive (TP) refers to the number of positive samples correctly identified as positive by the model. False Negative (FN) refers to the number of positive samples incorrectly predicted as negative by the model. False Positive (FP) refers to the number of negative samples incorrectly predicted as positive by the model. True Negative (TN) refers to the number of negative samples correctly identified as negative by the model. C represents the area of the smallest rectangle that can enclose both boxes (A and B).

**Figure 12:**
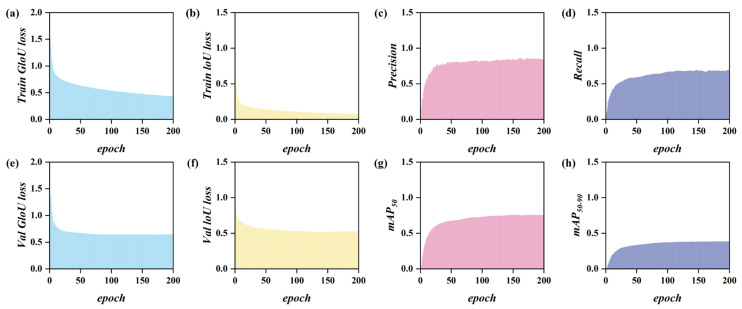
Training and validation convergence curve. (**a**) Train GIoU loss curve; (**b**) Train IoU loss curve; (**c**) precision curve; (**d**) recall curve; (**e**) Val GIoU loss curve; (**f**) Val IoU loss curve; (**g**) mAP_50_ curve; and (**h**) mAP_50–90_ curve.

Figure 12 illustrates the variation in loss function, precision, recall, mAP_50_, and mAP_95_ with respect to the number of training epochs for the TAR-DETR network. At epoch 25, the curve shows significant oscillation before stabilizing. From epoch 50 onwards, the accuracy of the TAR-DETR model is ensured. To further guarantee the accuracy of the TAR-DETR model, weight files generated during training are saved every 20 epochs for subsequent verification.

Early detection of most targets in complex urban traffic environments during the initial stages of an incident facilitates timely deployment of rescue operations. For the experiment’s reliability, a variety of algorithms, each with distinct design strategies, were chosen for comparative analysis. The selected methods include the two-stage object detection algorithm Faster R-CNN [48], the anchor-based single-stage object detection algorithms SSD [49] and YOLO v5n [50], and the anchor-free single-stage algorithms YOLO v8 and YOLO v10n [51]. The difference between YOLO v7 and v10n is in Appendix A. All algorithms were evaluated under the same experimental conditions, and Table 2 presents the results.

**Figure 13:**
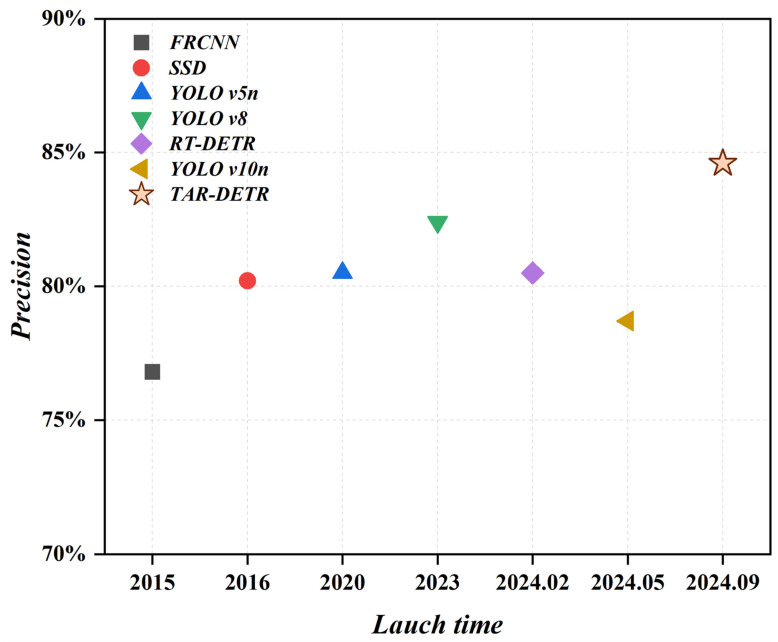
Compared to previously advanced real-time object detectors, our TAR-DETR achieves state-of-the-art performance.

Among all the algorithms, TAR-DETR achieved the highest mAP_50_ and various multi-scale detection scores (precision, mAP_50_, mAP_95_), further validating its strong multi-scale representation capability. Figure 13 shows the relationship between the launch time of different models and the magnitude of mAP_50_. Newer models do not necessarily outperform older ones. For instance, the accuracy of YOLO v10n is comparable to that of YOLO v5, but not as good as YOLO v8, highlighting the need for specialized models trained to complex traffic environments.

An analysis of Table 3 shows that the baseline + CGRFN model achieves a 0.3% increase in average accuracy for detecting urban traffic objects compared to the original RT-DETR. Although the model shows a slight increase in parameters and size, it achieves better accuracy in certain behavioural categories, maintains overall performance, reduces computational costs, and improves its practical applicability in real-world scenarios. When the FBM and DIF modules were integrated into CGRFN, the average accuracy of identifying targets improved by 1.3%.

**Figure 14:**
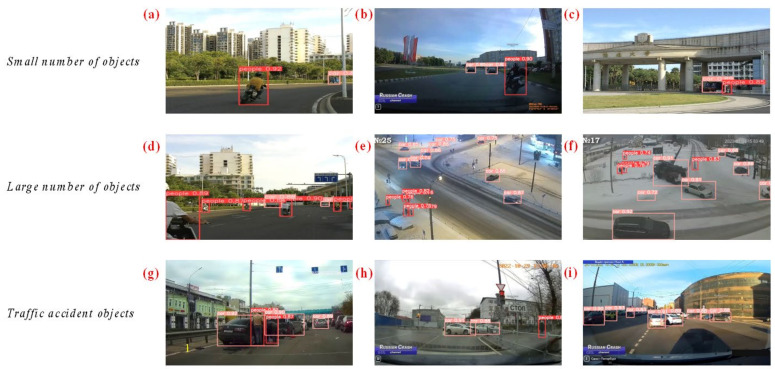
Object detection results of TAR-DETR. (**a**–**c**) Small number of objects; (**d**–**f**) large number of objects; (**g**–**i**) different traffic crash objects.

Urban traffic crashes exhibit significant stochasticity in terms of location, time, and environmental factors. As depicted in Figure 14, the anti-jamming ability of TAR-DETR under various conditions, including changes in lighting, obstacles, target quantity, and collision scenarios, is evaluated. As depicted in Figure 14a–f, TAR-DETR effectively detects objects under varying light conditions and object numbers at different times of day, night, and evening, successfully identifying most objects in the images. As depicted in Figure 14g–i, in collision scenarios, overlapping targets due to viewing angle issues make detection challenging. TAR-DETR captures global context in both horizontal and vertical directions through spatial feature reconstruction by the CGRFN, successfully modelling the rectangular key region to detect people–car collisions. Additionally, TAR-DETR successfully overcomes interference caused by changes in viewing angle, making it suitable for applications such as road surveillance, UAVs, and driver’s seat cameras.

### 4.2. Analysis of Extracted Traffic Crash Risk Features

**Figure 15:**
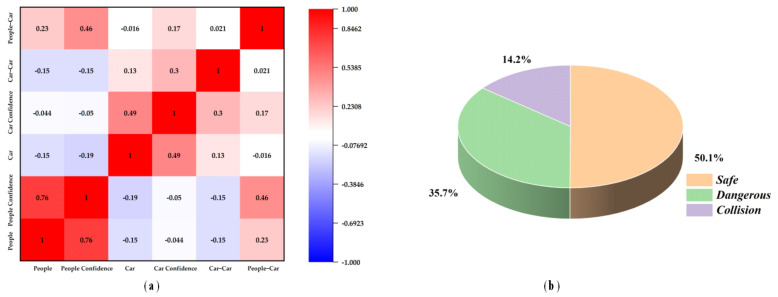
Analysis of TAR–2 traffic crash risk dataset. (**a**) Correlation matrix for TAR–2 dataset; (**b**) percentage of different categories in TAR–2 dataset.

Figure 15a shows the correlation matrix between features. Each individual cell denotes the magnitude and orientation of the linear correlation between two characteristics, encompassing a scale from −1 to 1. A value of 1 (expressed in red diagonals) indicates a complete positive correlation. A red shadow indicates positive correlation, indicating that when one feature increases, the other also increases. Conversely, a blue shadow represents negative correlation, signifying that as one feature increases, the other decreases. Numerous off-diagonal values show moderate to strong positive correlations (ranging from 0.4 to 0.8), indicating that some features are highly interrelated and may provide overlapping information. For example, features such as people confidence, people–people, and car–car, car confidence exhibit strong positive correlations. Some features (such as those positioned above the matrix) exhibit marginally reduced correlations, suggesting a greater degree of autonomous contributions to the dataset.

In addition, several characteristics exhibited moderate correlations, providing a wider range of information. However, no significant negative correlations were observed within the matrix. The lack of robust negative correlations and close-to-zero correlations also emphasizes the possibility of redundancy among the chosen features. This may be because the share of crash scenarios in the TAR-2 dataset is relatively small at 14.2%, resulting in insufficient positive correlations among car–car and people–car interactions, which leads to their relatively independent contribution to the dataset.

### 4.3. Online Inference of Traffic Crash Risk Based on WOA-SA-SVM

**Figure 16:**
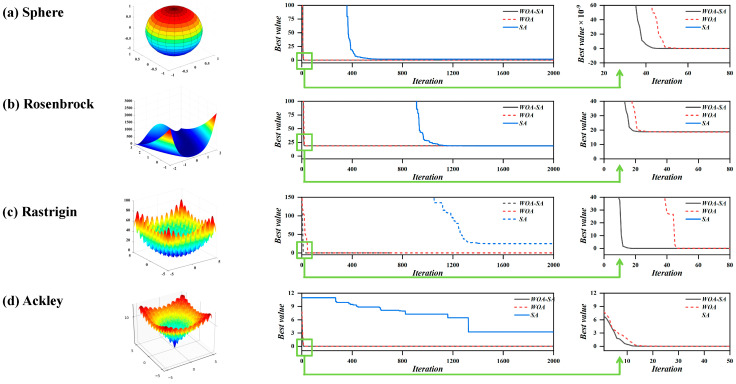
WOA-SA solution results for four typical test functions. (**a**) Sphere; (**b**) Rosenbrock; (**c**) Rastrigin; (**d**) Ackley.

To assess the optimization effectiveness of WOA-SA, it is employed for addressing the minimum value challenges posed by four representative test functions. Table 4 presents the details of the test functions and their respective ranges, including two unimodal functions and two multimodal functions. The algorithm’s performance is enhanced with a decrease in the solution’s value. The number of iterations allows for the observation of variations in convergence speed and precision. In the simulation test, the number of iterations was set to 2000, dimension D to 20, population size to 200, initial temperature to 100, and cooling rate to 0.95. Figure 16 shows the optimization results for different test functions.

Figure 16 illustrates that the final convergence accuracy of WOA is nearly identical to that of WOA-SA. However, zooming in on the function image reveals that WOA-SA has a clear advantage in convergence speed, while SA excels at escaping local optima. The SA optimization algorithm effectively escapes local optimal, whereas WOA demonstrates strong global search capability, resulting in faster convergence. Due to t, the complementary advantages of the optimization algorithms, WOA-SA excels in both convergence accuracy and speed. WOA-SA integrates the global search ability of WOA with enhanced optimization efficiency, while the inclusion of WOA improves the capacity to avoid local optima. Thus, the hybrid bionic optimization algorithm WOA-SA incorporates the distinct characteristics of individual algorithms.

The dataset for traffic collision risk features is split into a training set of 1332 samples and a test set of 333 samples, amounting to a total of 1665 samples. The accuracies of SVM, WOA-SVM, SA-SVM, and WOA-SA-SVM were compared. The penalty factor for the SVM is configured as 100, with the kernel function parameter set to 1. The model utilizes the C-SVC type and the RBF kernel function. The parameters of the WOA-SVM, SA-SVM, and WOA-SA-SVM detection and identification models are set as follows: (1) the number of whales is 200, (2) the optimization dimension (dim) is 2.0, (3) the penalty factor is searched in the range C = [1, 100], and (4) the RBF parameter is searched in the range g = [0.001, 1].

As depicted in Table 5, the accuracy of SVM for traffic crash risk inference is 60.96%, WOA-SVM is 65.76%, and SA-SVM is 62.21%. However, the inference accuracy of WOA-SA-SVM is 68.17%. This demonstrates that intelligent optimization algorithms can substantially enhance the inference accuracy of SVM when applied to the same dataset. WOA-SA-SVM has higher traffic crash risk prediction accuracy than WOA-SVM and SA-SVM. The WOA-SA-SVM has fewer support vectors, leading to a more straightforward structure and lower complexity in creating a high-dimensional linear classification surface. Despite having a higher penalty factor in its parameter configuration, SVM’s inference accuracy is notably lower than that of WOA-SA-SVM. In the same dataset, WOA-SA-SVM outperforms SVM by 7.21% in inference accuracy.

In our study, “detection time” refers to the duration needed for the model to complete image recognition tasks, whereas “inference time” denotes the time required to analyze and estimate the likelihood of traffic collisions. As shown in Table 6, both detection and inference times are consistently maintained within the millisecond range, highlighting the model’s high efficiency. Regarding the balance between real-time performance and accuracy, results from ten instances labelled “Crash” demonstrate that despite the rapid recognition and inference processes, the model effectively determines the correct outcomes, maintaining a strong balance between accuracy and performance.

**Figure 17:**
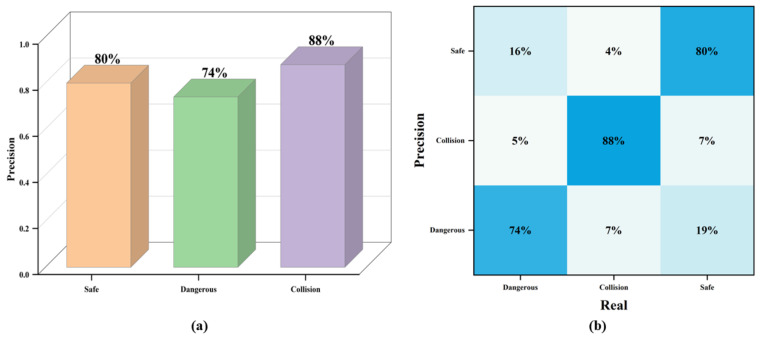
The inference results of TAR-2. (**a**) The precision of inference for each of the three categories; (**b**) the confusion matrix for WOA-SA-SVM.

The complete inference of the TAR-2 dataset using WOA-SA-SVM has an average accuracy of 80%, with class-wise accuracies of 80% for “Safe”, 74% for “Dangerous”, and 88% for “Collision”, as shown in Figure 17a. To further investigate the disturbing factors in the inference model, the confusion matrix is plotted, as shown in Figure 17b. In the inference processes, 16% of the “Dangerous” categories were incorrectly inferred as “Safe”. This may be due to the lack of overlapping “car-to-car” or “person-to-car” object frames in most of the “Dangerous” instances, leading to poor generalization of the distance feature, which results in errors. At the same time, this may explain the reason behind the misclassification of 19% of the “Safe” class instances as “Dangerous”.

Although WOA-SA-SVM’s inference accuracy for “Collision” classes is very high, with only 4% misclassified as “Safe” and 7% as “Dangerous”, there is still a need to analyze the misclassified instances. Misclassification of “Collision” inference can lead to significant economic and life losses.

**Figure 18:**
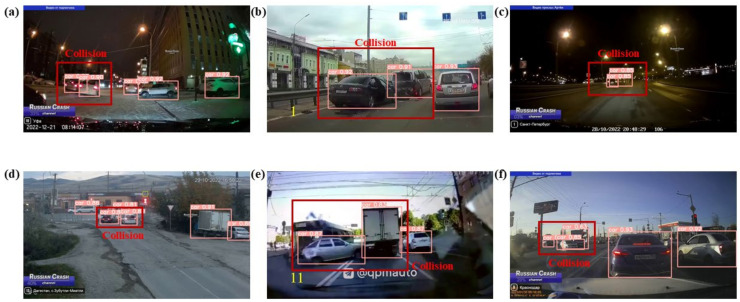
The instances of wrong inference (“Collision” to “Dangerous”). (**a**–**f**) Collision instances are inferred as dangerous in various environments.

**Figure 19:**
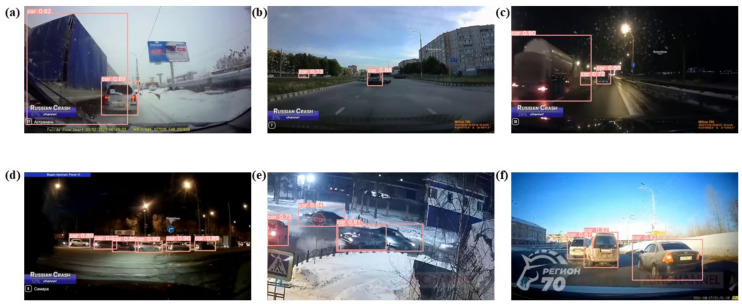
The instances of wrong inference (“Collision” to “Safe”). (**a**–**f**) Collision instances are inferred as safe in various environments.

As depicted in Figure 18, the instance that should have been inferred as “Collision” was incorrectly referenced as “Dangerous”. The location of the “Collision” is highlighted with a red box for clear observation. Due to the complexity of the collision environment, a larger number of objects can be observed in a scene at the same time, and the inference model observes a change in distance and number first ruling out the “Safe” class. Most incorrect classifications from “Collision” to “Dangerous” are due to inadequate inference of different car distances in congested traffic. In this study, only the shortest car-to-car distance was utilized as a feature, and no features were extracted for car-to-car distances within a specific area. This may have contributed to the inference error. As depicted in Figure 19, the instance that should have been inferred as “Collision” was incorrectly referenced as “Safe”. As depicted in Figure 19b, it is observed that the car disintegrates after the crash, and TAR-DETR fails to identify the disintegrated parts, indicating that feature extraction for the disintegrated parts was not considered, contributing to inference errors. This disintegration phenomenon frequently occurs in real crashes. This creates a paradox in a two-dimensional image, where “Collision” happens but the distance appears large. This error is due to the proximity of surveillance viewpoints and the conversion from 3D to 2D images.

## 5. Discussion

The findings of this study provide valuable insights for improving urban traffic safety. By accurately identifying traffic crash risks in real-time, the proposed models can enable prompt interventions, thereby reducing crashes and fatalities related to traffic. This approach could be instrumental in the development of more advanced traffic management systems that predict and mitigate risks before they intensify.

Still, there are some limitations of the study. The performance of TAR-DETR is significantly affected by reduced image quality, which impairs feature extraction and reduces detection accuracy—critical in high-precision applications such as autonomous driving and security surveillance. The CGRFN model, particularly the PCE and FBM components, faces challenges under adverse weather conditions like heavy rain or fog. Noise and blur diminish visual contrast and edge details, complicating target differentiation from the background for modules such as RCM. This impairs effective feature representation, leading to reduced detection performance. In low-light conditions, TAR-DETR’s ability to detect and localize small or blurred objects is impaired by noise and reduced visual features. Similarly, the multi-layer feature pyramid in CGRFN may fail to extract and enhance features effectively, resulting in substantial information loss. Poor signal-to-noise ratios during feature fusion further reduce inter-layer feature complementarity, thereby impacting detection accuracy, especially for small targets.

The TAR-2 dataset reveals missing negatively correlated features, which weakens the model’s generalization ability. Additionally, the TAR-2 dataset has a low percentage of the “Collision” category, which may limit the inference of all crash types. Analysis of the WOA-SA-SVM inference results shows that some “Collision” instances are misclassified as “Dangerous” due to the lack of car distance features in a potential area. Furthermore, some “Collision” instances are incorrectly classified as “Safe”, caused by the proximity of surveillance viewpoints and the conversion from 3D to 2D images. Furthermore, some “Collision” instances are incorrectly classified as “Safe” due to the proximity of surveillance viewpoints and the conversion from 3D to 2D images. In response to these issues, we will focus on improving the model by incorporating more crash examples to balance the dataset and adding additional traffic crash features, such as car distance and multi-view data, to enhance feature representation. Specifically, we plan to integrate advanced sensors or multi-source fusion techniques to obtain accurate vehicle spacing information, which is essential for distinguishing between hazardous and collision scenarios. Additionally, efforts will be made to mitigate the effects of converting 3D information into 2D space by either incorporating 3D data directly or using deep learning models capable of processing 3D spatial relationships. Rigorous scenario-based testing, especially in high-risk environments, will be conducted to ensure the model’s robustness in real-world applications. We are exploring the use of stereo cameras, along with an alternative method involving coordinate transformation and projective transformation, to enhance distance estimation [53]. Moreover, we are exploring mechanisms for real-time feedback to flag uncertain cases for manual review, thus reducing the risk of critical misclassifications that could lead to loss of life.

Future research should incorporate a larger dataset of crash examples and additional traffic risk features, such as Proportion of Stopping Distance (PSD), Mean-Time To Resolution (MTTC), and Post Encroachment Time (PET), to enable a more comprehensive analysis of traffic crash risk. Attention should also be given to the conversion of distances from 2D space to reduce inference errors. Images of car disintegration resulting from traffic crashes still need to be collected.

## 6. Conclusions

This paper proposes an online inference method for urban traffic crash risk, utilizing the self-developed TAR-DETR and WOA-SA-SVM models. The method aims to assess real-time traffic crash risk, thereby reducing loss of life and property in complex urban traffic environments. The main conclusions are as follows:

1. The TAR-1 urban traffic objects dataset, consisting of 1665 images, was self-developed by combining traffic information from major roads around Hainan University with Russian car crash news. Feature extraction from TAR-1 led to the creation of the TAR-2 urban traffic crash risk dataset, which includes six variables and three classifications: “Safe” (50.1%), “Dangerous” (35.7%), and “Collision” (14.2%).
2. The proposed TAR-DETR aims to improve object detection accuracy in complex urban traffic environments with varying illumination, obstacles, object counts, and collision scenarios. To address object overlap and image edge target extraction, a Con-text-Guided Reconstruction Feature Network (CGRFN) is introduced. Experimental results show that the proposed model achieves 76.8% mean average precision (mAP_50_) on the TAR-1 dataset. Compared to the original algorithm, it achieves a 3.7% increase in precision (P). The proposed method outperforms other state-of-the-art models and effectively handles multi-object detection in complex urban traffic environments, providing valuable technical support for extracting traffic risk features from TAR-1.
3. An online inference method based on WOA-SA-SVM is proposed. A new hybrid intelligent optimization algorithm, WOA-SA, is developed to address the limitations of single intelligent optimization algorithms. The key parameters (C, g) of the SVM are optimized using WOA-SA, enabling effective risk assessment of urban traffic crashes. The mean accuracy of inference is 80%.

## Figures and Tables

**Table 1:** Format of content components of TAR-2.

Description	Value
Number of rows	1665
Number of columns	6
Categorical variables	3

**Table 2:** Comparative experiments of TAR-DETR and other methods.

Algorithm	Precision	Recall	mAP_50_	mAP_95_
SSD	76.8%	60.3%	62.5%	30.9%
F-RCNN	80.2%	65.4%	72.8%	37.9%
YOLO v5n	80.5%	64.4%	71.3%	37.4%
YOLO v8	82.4%	64.4%	72.2%	37.9%
RT-DETR	80.5%	68.1%	73.1%	37.2%
YOLO v10n	78.7%	63.5%	71.8%	37.7%
TAR-DETR	84.6%	70.0%	76.8%	39.4%

**Table 3:** Ablation experiment results of TAR-DETR.

Algorithm	Precision	Recall	mAP_50_	mAP_95_
baseline	80.1%	70.09%	73.1%	37.2%
baseline + CGRFN	81.8%	69.12%	75.3%	38.4%
baseline + CGRFN + FBM + DIF	83.1%	63.32%	76.8%	39.7%

**Table 4:** Four typical functions [52] were used to test the WOA-SA algorithm.

Test Function	Expression	Parameter Value	Function Types
Sphere	$f(x)=\sum_{i=1}^{n}x_{i}^{2}$	50 ≤ *x*_i_ ≤ 100	Unimodal function
Rosenbrock	$f(x)=\sum_{i=1}^{n-1}\left[100{\left(x_{i+1}-x_{i}^{2}\right)}^{2}+{\left(x_{i}-1\right)}^{2}\right]$	10 ≤ *x*_i_ ≤ 25	Unimodal function
Rastrigin	$f(x)=10n+\sum_{i=1}^{n}\left[x_{i}^{2}-10\cos\left(2\pi x_{i}\right)\right]$	2.56 ≤ *x*_i_ ≤ 5.12	Multimodal function
Ackely	$f(x)=-20\exp\left(-0.2\sqrt{\frac{1}{n}\sum_{i=1}^{n}x_{i}^{2}}\right)-\exp\left(\frac{1}{n}\sum_{i=1}^{n}\cos\left(2\pi x_{i}\right)\right)+20+e$	−5 ≤ *x*_i_ ≤ 5	Multimodal function

**Table 5:** Important parameters and traffic crash risk inference results.

Model	SVM Parameters (C, g)	Total Number of Support Vectors	Accuracy (%)
SVM	(100, 1)	78	60.96
WOA-SVM	(70.36, 0.76)	148	65.76
SA-SVM	(99.81, 0.03)	333	62.21
WOA-SA-SVM	(82.22, 0.64)	128	68.17

**Table 6:** Detection time of TAR-DETR and inference time of WOA-SA-SVM.

Number of People	Number of Cars	Detection Time	Inference Time	Result
1	4	18.9 (ms)	0.996 (ms)	Crash
0	3	18.9 (ms)	0.996 (ms)	Crash
1	2	19.7 (ms)	0.829 (ms)	Crash
0	12	19.9 (ms)	0.996 (ms)	Crash
0	3	18.9 (ms)	0.996 (ms)	Crash
1	3	19.9 (ms)	0.996 (ms)	Crash
1	2	18.9 (ms)	0.829 (ms)	Crash
1	3	21.9 (ms)	0.997 (ms)	Crash
0	8	21.9 (ms)	0.996 (ms)	Crash

## Data Availability

The dataset is available from the first author. However, only video formats are available (labels and photo are not available due to laboratory restrictions).

## Nomenclature

Abbreviation	Description
CGRFN	Context-Guided Reconstruction Feature Network
WOA	Whale Optimization Algorithm
SA	Simulated Annealing
SVM	Support Vector Machine
WHO	World Health Organization
YOLO	You Only Look Once
USV	Unmanned Aerial Vehicle
DETR	Detection Transformer
RT-DETR	Real-Time Detection Transformer
RF	Random Forest
DJ	Decision Jungle
L-GBM	Light Gradient Boosting Machine
CatBoost	Categorical Boosting
MVNB	Multivariate Negative Binomial
DSA	Dynamic Spatial Attention
GRU	Gated Recurrent Unit
RCM	Rectangular Self-Calibration Module
PCE	Pyramid Context Extraction
FBM	Fuse Block Multi-Module
DIF	Dynamic Interpolation Fusion
TAR-1	Urban Traffic Objects Dataset
TAR-2	Urban Traffic Rrash Risk Features Dataset
SSM	Surrogate Safety Measures
TAR-DETR	Traffic Crash Risk Detection Transformer
CA	Coordinated Attention
BN	Batch Normalization
ReLU	Rectified Linear Unit
EDT	Integrated Decision Tree
KNN	K-Nearest Neighbor
KNNs	K-Nearest Neighbors
ANNs	Artificial Neural Networks
AP	Average Precision
IoU	Intersection over Union
PR	Precision-Recall
GIoU	Generalized Intersection over Union
TP	True Positive
TN	True Negative
FN	False Negative
HSD	Honest Significant Difference
HOA	Hiking Optimization Algorithm
TwoArchHHO	Two-Archive Harris Hawk Optimization
MaOOPF	Many-Objective Optimal Power Flow
APO	Artificial Protozoa Optimizer

## Appendix A. Differences Between YOLO v7, YOLO v8, and YOLO v10n

YOLOv8 simplifies the network structure by introducing the EfficientRep backbone, which reduces the number of parameters and computational costs while maintaining performance. YOLOv8 improves the feature fusion methods of PANet and FPN by introducing a new Path Aggregation Network (Rep-PAN), enhancing the fusion of multi-scale features. Compared to the PANet structure in YOLOv7, Rep-PAN enhances target detection robustness without adding complexity, thanks to a more efficient path aggregation strategy. YOLOv8 introduces an improved version of CIoU (Complete Intersection over Union) to enhance the accuracy of the target box position. Compared to YOLOv7, YOLOv8′s loss function better handles the shape, size, centre position, and scale of the bounding box. YOLOv8 continues to refine the Anchor-Free mechanism, improving generalization when detecting targets at different scales. YOLOv8 introduces new training strategies, including Mosaic Augmentation, MixUp Augmentation, and Self-Adversarial Training, which enhance data augmentation diversity and improve model generalization. These enhancements allow YOLOv8 to maintain high detection performance even with limited data.

YOLO v10n introduces several improvements over YOLO v8, including NMS-Free Training, which eliminates the need for non-maximum suppression, thereby reducing inference latency. The model includes holistic design optimizations such as lightweight classification heads and spatial-channel decoupled down sampling. Additionally, YOLOv10 integrates large-kernel convolutions and partial self-attention modules to enhance performance without significantly increasing computational costs.

## Appendix B. The Basic Principles of SVM

SVM uses nonlinear mapping to transform data from a low-dimensional space to a higher-dimensional feature space, where it constructs an optimal hyperplane for classification [52]. SVM addresses the binary classification problem by finding a hyperplane L, which maximizes the distance between the sampling points of different categories. The fundamental steps involved in SVM are as follows [45]:

Step 1: *L*_1_ and *L*_2_ are defined as hyperplanes, as shown in Equation (A1).

(A1) $$\left\{\begin{array}{c} L_{1}:w\cdot x=+1\\ L_{2}:w\cdot x=-1 \end{array}\right.$$

where *ω* is the weight vector; the distances from *L* to *L*_1_ and *L*_2_ are *d*_1_ and *d*_2_, respectively. And *d*_1_ = *d*_2_. The margin distance between the two lines can be calculated using Equation (A2).

(A2) $$M\arg in=2d_{1}=2\frac{\left|Ax_{0}+By_{0}+C\right|}{\sqrt{A^{2}+B^{2}}}=2\frac{\left|w\cdot x+b\right|}{\left\|w\right\|}=\frac{2}{\left\|w\right\|}$$

when the margin is largest, the samples in the training set *D* = {(*x*_i_, *y*_i_), i = 1, 2, …, n} are

(A3) $$y_{i}\left[\left(w\cdot x_{i}\right)+b\right]-1\geq 0$$

where *i* = 1, 2, …, *n*. *y*_i_ = ±1.

Step 2: Construct the optimal hyperplane.

(A4) $$\left\{\begin{array}{c} \min\frac{1}{2}{\left\|w\right\|}^{2}=\min\frac{1}{2}w^{T}w\\ s.t.y_{i}\left[\left(w\cdot x_{i}\right)+b\right]-1\geq 0 \end{array}\right.$$

Step 3: The Lagrangian function is introduced to obtain

(A5) $$L\left(w,b,\alpha \right)=\frac{1}{2}w^{T}w-\sum_{i=1}^{n}\alpha_{i}\left\{y_{i}\right.\left[\left(w\cdot x_{i}\right)+b\right]-\left.1\right\}$$

Step 4: Calculate the partial derivatives of *w* and *b* in Equation (A5), set them equal to 0, and solve the resulting equations to obtain

(A6) $$L\left(w,b,\alpha \right)=\sum_{i=1}^{n}\alpha_{i}-\frac{1}{2}\sum_{i=1}^{n}\alpha_{i}y_{i}\sum_{j=1}^{n}\alpha_{i}y_{i}\left(x_{i}\cdot x_{j}\right)$$

The constraint condition is α_j_ ≥ 0 and $\sum_{i=1}^{n}\alpha_{i}y_{i}=0$. After solving the optimal solutions *α**, *b**, *w**, the optimal function, as shown in Equation (A1), can be derived.

(A7) $$h\left(x\right)=\mathrm{sgn}\left(\left(w^{*}x\right)+b^{*}\right)=\mathrm{sgn}\left(\sum_{j=1}^{n}{\alpha^{*}}_{i}y_{i}\left(x_{i}\cdot x\right)+b^{*}\right)$$

where *x* represents the test sample and *x*_i_ (*i* = 1, 2, …, *n*) represents all the training samples.

Step 5: In Equation (A3), the relaxation factor ξ is introduced to relax the constraint conditions. Then, *y*_i_[(*ω*·*x*_i_) + b] − 1 + ξ ≥ 0, where 0 < 1. The penalty factor *C* is introduced in Equation (A4) to make the value of *ξ* as small as possible where *C* is a constant. And greater than 0.

(A8) $$\min\frac{1}{2}{\left\|w\right\|}^{2}+C\sum_{i=1}^{n}\xi_{i}=\min\frac{1}{2}w^{T}w+C\sum_{i=1}^{n}\xi_{i}$$

However, the linear classification of SVM cannot be realized in an accurate traffic crash risk sample. Therefore, the nonlinear mapping function is adopted to meet the requirements of the SVM.

Step 6: Data points will be transformed into different spaces through the introduction of additional mapping functions. In this case, the constraint condition of the original feature is

(A9) $$y_{i}\left[\left(w\cdot \phi \left(x_{i}\right)\right)+b\right]-1+\xi \geq 0$$

The objective function *Φ*(*x*_i_) is

(A10) $$\underset{w,b}{\min}\frac{1}{2}w^{T}w+C\sum_{i=1}^{n}\xi_{i}$$

Step 7: The Lagrangian function is constructed by Equation (A10).

(A11) $$L\left(w,b,\alpha ,\beta ,\xi \right)=\frac{1}{2}w^{T}w+C\sum_{i=1}^{n}\xi_{i}-\sum_{i=1}^{n}{\alpha^{*}}_{i}\left[y_{i}\left[\left(w\cdot \phi \left(x_{i}\right)\right)+b\right]-1+\xi \right]-\sum_{i=1}^{n}\beta_{i}\xi_{i}$$

Step 8: The partial derivatives of *w*, *b*, *ξ* of Equation (A11) are obtained, respectively. And the partial derivative result is equal to 0. Substitute the calculation results into Equation (A11).

(A12) $$L\left(w,b,\alpha ,\beta ,\xi \right)=\sum_{i=1}^{n}\alpha_{i}-\frac{1}{2}\sum_{i=1}^{n}\sum_{j=1}^{n}\alpha_{i}\alpha_{j}y_{i}y_{j}\left[\phi \left(x_{i}\right)\cdot \phi \left(x_{i}\right)\right]$$

By the Karush—Kuhn—Tucker (KKT) conditions, the optimal solution must satisfy the following criteria:

(A13) $$\left\{\begin{array}{c} y_{i}\left[w\cdot \phi \left(x_{i}\right)+b\right]-1+\xi_{i}=0\\ \beta_{i}\xi_{i}=0\to \left(C-\alpha_{i}\right)\xi_{i}=0 \end{array}\right.$$

Step 9: The kernel function K (*x*_i_, *x*_j_) is used to replace *Φ*(*x*_i_)·*Φ*(*x*_i_) in Equation (A6). The SVM model is

(A14) $$\left\{\begin{array}{c} \max\sum_{j=1}^{n}\alpha_{i}-\frac{1}{2}\sum_{i=1}^{n}\alpha_{i}\alpha_{j}y_{i}y_{j}K\left(x_{i},x_{j}\right)\\ s.t.\sum_{i=1}^{n}\alpha_{i}y_{i}=0\\ s.t.0\leq \alpha_{i}\leq C,i=1,2,3,\cdots ,n \end{array}\right.$$

Step 10: The optimal classification function can be obtained

(A15) $$f\left(x\right)=\mathrm{sgn}\left[\left(w\cdot \phi \left(x\right)\right)+b\right]=\mathrm{sgn}\left[\sum_{i=1}^{n}\alpha_{i}\alpha_{j}K\left(x_{i},x\right)+b\right]$$

## Appendix C. Whale Optimization Algorithm

The WOA is an optimization technique based on the hunting behaviour of humpback whales [46]. It simulates the unique hunting method of humpback whales using “bubble nets” and employs strategies such as encircling prey, spiralling, and random search to find the global optimal solution. The algorithm gradually approaches the optimal solution by updating individual positions within the population, demonstrating strong global search and local optimization capabilities.

The whale’s search range encompasses the global solution space, requiring it to determine the location of its prey to encircle it. The formula is as follows [46]:

(A16) $$D=\left|C\cdot X^{*}\left(t\right)-X\left(t\right)\right|$$

(A17) $$X\left(t+1\right)=X^{*}\left(t\right)-A\cdot D$$

where *t* is the current iteration; *X*^∗^ is the best position obtained so far; and *A* and *C* represent the coefficient vectors.

(A18) $$A=2a\cdot r_{1}-a$$

(A19) $$C=2\cdot r_{2}$$

where *a* is the convergence factor, which falls linearly from 2 to 0 as the number of iterations rises, as shown in Formula (A20). And *r*_1_ and *r*_2_ represent random vectors between [0, 1].

(A20) $$a=2-\frac{2t}{t_{\max}}$$

where *t*_max_ is the max iterations.

By applying the logarithmic spiral method to update positions, a new location can be identified during bubble net feeding that follows the whale’s spiral motion, linking its initial position with the prey’s current location. The following describes its mathematical model:

(A21) $$X\left(t+1\right)=D^{\prime }\cdot e^{bl}\cdot \cos\left(2\pi l\right)+X^{*}\left(t\right)$$

(A22) $$D^{\prime }=\left|X^{*}\left(t\right)-X\left(t\right)\right|$$

where *D*′ is the distance between the current searching individual and the target prey; *b* is an internal parameter controlling the logarithmic spiral shape; and *l* is a random number between [−1, 1].

The WOA’s location update formula is as follows: the chance *p* of selecting either the bubble net feeding technique or the above encircling prey is 0.5.

(A23) $$X\left(t+1\right)=\left\{\begin{array}{c} X^{*}\left(t\right)-A\cdot D,p<0.5\\ D^{\prime }\cdot e^{bl}\cdot \cos\left(2\pi l\right)+X^{*}\left(t\right),p\geq 0.5 \end{array}\right.$$

where *p* is a random digit in the interval [0, 1].

## Appendix D. Simulated Annealing Algorithm

The SA algorithm is a stochastic search algorithm for global optimization problems, inspired by the annealing process of solids [47]. By simulating the gradual convergence of atomic arrangements to the lowest energy state during cooling, SA performs a random search in the solution space and can escape local optima. It allows acceptance of inferior solutions with a certain probability, thus avoiding premature convergence to a local optimum.

Assuming that the objective function needs to be minimized, an initial solution (*X*_0_) and an initial temperature (T_0_) are first set. In each iteration, a new candidate solution is generated, and the decision to accept it is based on a probability function. The updated formula for this process is as follows [47]:

(A24) $$x_{new}=x_{current}+\Delta x$$

(A25) $$P=exp\left(-\frac{\Delta E}{T}\right)$$

where X_current_ is the current location of the solution; Δx is the amount of variation, which is usually random, used to explore the solution space; ΔE represents the energy difference (or cost function difference); and *T* represents the current temperature. This probability allows the algorithm to potentially accept a worse solution than the current one, thereby enabling exploration of a wider solution space and avoiding local optima.

The temperature T progressively decreases with each iteration, and a common cooling function is given as follows:

(A26) $$T=\alpha \times T_{0}$$

where T is the initial temperature and *α* is the cooling rate (a constant less than 1). As the iterations continue, the temperature gradually decreases, thereby decreasing the likelihood of accepting a suboptimal solution. This gradual reduction in temperature allows for the algorithm to converge towards an optimal or near-optimal solution.

## Appendix E. Hybrid Bionic Intelligent Optimization Algorithm

The Hybrid Bionic Intelligent Optimization Algorithm steps are as follows:

Step 1: The dataset is first loaded, then split into training and testing sets, and finally normalized.

Step 2: The optimal SVM parameters are determined by combining the Whale Optimization Algorithm (WOA) with Simulated Annealing (SA). The fitness values are calculated by the WOA-SA.

Step 3: Compare and select the best fitness values obtained by the two algorithms.

Step 4: The algorithm is iterated continuously. The WOA and SA are used iteratively to calculate the fitness value in their respective subpopulations.

Step 5: Repeat Step 3 to determine whether the maximum number of iterations or preset conditions are met. If the end condition is reached, the algorithm ends. Otherwise, repeat Step 4.

Step 6: Output the optimal parameters (C, g) to the SVM model. WOA facilitates global exploration, while SA helps avoid local optima, optimizing the SVM parameters c and g. Finally, the SVM model is trained using the training set and evaluated on the test set to assess its accuracy and generalization.

The SVM algorithm shows high resilience and the ability to generalize well with small sample data, especially in cases of nonlinearity. However, its classification accuracy is highly dependent on the selection of g and C. To enhance the precision of SVM in predicting the risk of urban traffic crashes, the parameters (C, g) can be effectively optimized by incorporating Bionic Intelligent Optimization Algorithms. The WOA (Whale Optimization Algorithm) and SA (Simulated Annealing) algorithms were selected and combined to meet the needs of traffic risk event identification. The WOA demonstrates quicker convergence and improved optimization accuracy during the initial phases, but it has a tendency to become trapped in local optima as it progresses, leading to slower convergence. SA, on the other hand, can effectively overcome this limitation but is characterized by numerous parameters and high computational cost. Therefore, integrating WOA and SA into a new Hybrid Bionic Intelligent Optimization Algorithm can improve overall optimization performance.
